# Long-term outcomes of children treated for Cushing’s disease: a single center experience

**DOI:** 10.1007/s11102-016-0756-8

**Published:** 2016-09-27

**Authors:** Galina Yordanova, Lee Martin, Farhad Afshar, Ian Sabin, Ghassan Alusi, Nicholas P. Plowman, Fiona Riddoch, Jane Evanson, Matthew Matson, Ashley B. Grossman, Scott A. Akker, John P. Monson, William M. Drake, Martin O. Savage, Helen L. Storr

**Affiliations:** 1 Department of Pediatrics and Medical Genetics, MU-Varna, Varna, Bulgaria; 2 Department of Pediatric Endocrinology, Royal London Hospital, Whitechapel Road, Whitechapel, London, E1 1BB UK; 3 Departments of Neurosurgery, St Bartholomew’s Hospital, West Smithfield, London, EC1A 7BE UK; 4 Departments of Otolaryngology, St Bartholomew’s Hospital, West Smithfield, London, EC1A 7BE UK; 5 Departments of Radiotherapy, St Bartholomew’s Hospital, West Smithfield, London, EC1A 7BE UK; 6 Departments of Clinical Biochemistry, St Bartholomew’s Hospital, West Smithfield, London, EC1A 7BE UK; 7 Departments of Radiology, St Bartholomew’s Hospital, West Smithfield, London, EC1A 7BE UK; 8 Oxford Centre for Diabetes, Endocrinology and Metabolism, University of Oxford, Oxford, OX3 7LJ UK; 9 Centre for Endocrinology, William Harvey Research Institute, Barts and the London School of Medicine and Dentistry, Queen Mary University of London, First Floor, John Vane Science Centre, Charterhouse Square, London, EC1M 6BQ UK

**Keywords:** Cushing’s disease, Pediatric, Outcome, Recurrence

## Abstract

**Purpose:**

Pediatric Cushing’s disease (CD) is rare and there are limited data on the long-term outcomes. We assessed CD recurrence, body composition, pituitary function and psychiatric comorbidity in a cohort of pediatric CD patients.

**Methods:**

Retrospective review of 21 CD patients, mean age at diagnosis 12.1 years (5.7–17.8), managed in our center between 1986 and 2010. Mean follow-up from definitive treatment was 10.6 years (2.9–27.2).

**Results:**

Fifteen patients were in remission following transsphenoidal surgery (TSS) and 5 were in remission following TSS + external pituitary radiotherapy (RT). One patient underwent bilateral adrenalectomy (BA). CD recurrence occurred in 3 (14.3 %) patients: 2 at 2 and 6 years after TSS and 1 7.6 years post-RT. The BA patient developed Nelson’s syndrome requiring pituitary RT 0.6 years post-surgery. Short-term growth hormone deficiency (GHD) was present in 14 patients (81 % patients tested) (11 following TSS and 3 after RT) and 4 (44 % of tested) had long-term GHD. Gonadotropin deficiency caused impaired pubertal development in 9 patients (43 %), 4 requiring sex steroid replacement post-puberty. Four patients (19 %) had more than one pituitary hormone deficiency, 3 after TSS and 1 post-RT. Five patients (24 %) had long-term psychiatric co-morbidities (cognitive dysfunction or mood disturbance). There were significant long-term improvements in growth, weight and bone density but not complete reversal to normal in all patients.

**Conclusions:**

The long-term consequences of the diagnosis and treatment of CD in children is broadly similar to that seen in adults, with recurrence of CD after successful treatment uncommon but still seen. Pituitary hormone deficiencies occurred in the majority of patients after remission, and assessment and appropriate treatment of GHD is essential. However, while many parameters improve, some children may still have mild but persistent defects.

## Introduction

Cushing’s disease (CD) is defined as hypercortisolism due to excess pituitary ACTH secretion by a corticotroph adenoma accounting for ~75 % of cases of pediatric Cushing’s syndrome [1]. The diagnostic and therapeutic strategies are largely based on experience in the adult CD population, although some differences in presentation and responses to therapy exist [2]. Long-term consequences of adult CD are well documented, including morbidity, recurrence and its sequelae [3], but limited data exist for pediatric patients after transsphenoidal surgery (TSS) and external pituitary radiotherapy (RT).

Growth and body composition are frequently compromised in pediatric CD at diagnosis, and following successful therapy, height and weight may not normalize [4, 5]. Early growth hormone treatment following remission of hypercortisolemia may improve catch-up growth and adult height [4]. Abnormal puberty with virilization and gonadotropin deficiency is recognized in CD [6]. Defects in pituitary function following remission of CD after TSS or RT are well described [7–9]. Hypercortisolemia also affects mood and may precipitate psychiatric disturbances, with uncertain long-term sequelae [3].

Due to the rarity of pediatric CD, only a few centers have experience of its treatment and follow-up with few published data on the long-term outcome following treatment during childhood, with most reports only looking at the short-term results. Differences in management protocols and definitions of remission or ‘cure’ have led to rates of successful treatment reported from 60 to 98 % [1, 8, 10, 11]. Variation is also seen for rates of recurrence after remission, ranging from 6 to 27 % [10, 12, 13]. We have previously extensively described the presenting features, diagnostic testing and early outcomes of children with CD: we now describe the longer-term outcome in 21 pediatric CD patients, diagnosed and treated in one center, concentrating on the prevalence of recurrence and features of linear growth, body composition, pubertal development, pituitary function and psychiatric status.

## Patients and methods

Twenty-one patients (13 males) were diagnosed with CD and treated at St. Bartholomew’s and The Royal London Hospitals, London, UK, between 1986 and 2010 (Table 1). Twelve patients were followed up at our center and 9 with endocrinologists in other institutions. Data were retrospectively collected and therefore there was some missing data. The mean length of follow-up from time of definitive therapy, which resulted in remission in all cases, was 10.6 years (2.9–27.2).

**Table 1:** Clinical features, height, BMI, pubertal status and baseline biochemical evaluation at diagnosis and latest assessment

Patient no.	Sex	FU interval (yr)	At diagnosis									At follow-up (latest assessment)			
			Age (yr)	Ht SDS	BMI SDS	Pubertal stage (TV: R/L)	00.00 h Cortisol (nmol/l)	09.00 h ACTH (ng/l)	Cortisol during LDDST (nmol/l) (% suppress)	Cortisol during HDDST (nmol/l) (% suppress)	CRH test (cortisol rise %)	Age^a^ (FU interval to latest height measurement^a^)	Ht SDS	BMI SDS	Pubertal development
1	F	2.9	14.7	−2.1	2.0	A2 B1 P4	983	24	588 (0)	–	81	19.8 (2.9)	**−2.84**	2.12	B1 P5 A2 M1
2	M	16.6	6.4	−1.6	5.1	G2 P2 (2/2)	285	26	184 (37)	<50	83	23.7 (16.6)	**−1.34**	0.01	Adult
3	M	15.7	13.7	−1.8	2.3	G3 P3 (3/3)	930	38	170 (65)	<50	87	29.6	–	–	Adult
4	M	17.0	17.8	−0.7	1.7	G4 (15/15)	287	66	41 (100)	<20	24	34.7 (7.6)	–	–	Adult
5	M	7.6	8.8	−1.1	3.2	G1 P1 (3/3)	552	61	265 (46)	–	35	1.0 (7.6)	**0.23**	1.46	Adult
6	M	5.3	6.4	−1.1	4.9	A1 G2 P3 (2/2)	490	48	601 (59)	–	2	12.3 (5.3)	0.32	2.71	P5 A2 (12/12)
7	M	5.9	13.2	−1.1	0.0	A2 G2 P3 (6/5)	1082	15	513 (51)	–	34	19.2 (4.4)	**−1.8**	0.52	Adult
8	M	9.9	8.2	−1.5	3.0	G2 P1 (2/2)	380	65	101 (84)	–	46	18.2 (9.9)	−0.51	0.99	TV 15/15
9	F	20.4	14.3	−0.3	2.3	B4 M0	216	13	369 (58)	79 (86)	117	34.7	–	–	Adult
10	F	27.2	16.4	−2.3	0.7	A2 B4 P4	460	53	250 (73)	<50	200	44.0	–	–	Adult
11	F	15.0	14.8	−2.9	0.8	B3 M0	478	25	842 (0)	<50	45	30.1 (11.2)	**0.00**	0.00	Adult
12	M	11.1	11.7	−1.8	3.1	G1 P2 (2/2)	222	15	55 (86)	<50	219	18.9 (7.3)	**−1.46**	−0.17	Adult
13	M	8.9	15.6	−1.1	3.0	A1 G4 P4 (12/12)	146	21	106 (77)	<50	80	24.7	–	–	Adult
14	M	13.6	16.8	−3.3	1.6	A3 G4 P4 (8/8)	586	52	370 (3)	<50	90	30.42 (3.9)	**−1.58**	−0.16	TV 12/12
15	F	11.4	13.8	−0.5	2.2	A3 B5 P5 M1	552	50	153 (78)	<50	6	25.4	–	–	Adult
16	M	8.1	6.6	−3.2	3.8	A1 G2 P2 (2/2)	857	33	695 (16)	207 (61)	268	15.1 (8.1)	−2.53	−1.90	TV 6/6
17	F	5.8	5.7	−0.2	6.9	A1 B1 P1	199	29	326 (38)	521 (0)	62	12.0 (5.8)	−0.03	1.12	B2 P1 A1 M1
18	F	5.7	14.1	−2.1	2.5	A3 B1 P4	611	96	22 (100)	–	59	19.9 (5.7)	**−1.22**	1.53	Adult
19	F	5.1	9.7	−0.4	2.1	A1 B1 P2	308	51	36 (94)	–	175	15.1 (5.1)	−0.53	−0.92	B4 P4 M1
20	M	5.2	12.9	0.1	2.2	A2 G2 P2 (2/2)	521	42	272 (53)	–	60	17.2 (2.6)	0.46	0.05	Adult
21	M	4.6	11.7	−1.6	2.7	A2 G1 P2 (2/2)	463	68	185 (43)	–	128	16.3 (4.6)	−1.99	1.41	Adult

FU interval, time interval from definitive therapy resulting in remission to latest assessment; Age^a^, age at final clinical assessment; FU interval to latest height measurement^a^, FU interval to latest height measurement, if different from the interval to latest clinical assessment; *Ht* height, Puberty staged by Tanner (16, 17). *B* breast development *G* genital stage, *P* pubic hair stage, *A* axillary hair stage, *M* menarche, *TV* testicular volume (ml), *R* right, *L* left, Serum cortisol 00.00 h (sleeping), *LDDST* low-dose dexamethasone suppression test (% cortisol suppression from baseline), *HDDST* high-dose dexamethasone suppression test (% cortisol suppression from baseline), *CRH* corticotrophin-releasing hormone. Height SDS at latest assessment, final (adult) heights are in bold

Informed consent was obtained and Institutional Review Board permission was granted for the release of anonymized data for publication.

### Definition of latest assessment

‘Latest assessment’ is defined as the time the individual patient was assessed most recently. This corresponds to the follow-up interval from definitive therapy (mean 10.6 years; range 2.9–27.2).

### Clinical features at diagnosis

Mean age at presentation was 12.1 years (5.7–17.8). The most common presenting features were weight gain (100 %), change in facial appearance (100 %), growth retardation (95 %). emotional lability (71 %) and fatigue (62 %). Other features included hypertension (43 %), acne (38 %), hirsutism (52 %), headaches (52 %) and striae (43 %). Less common presenting features were acute psychosis (Patient 1), hypokalemia (Patient 7), vertebral fractures due to severe osteoporosis (Patient 14) and anxiety combined with challenging behavior (Patient 17). Mean symptom duration prior to diagnosis was 1.8 years (range 0.5–3.5) (Table 1). At diagnosis, 8 patients (4 males) were pubertal, i.e. testicular volume ≥4 ml or breast stage ≥2 according to Tanner’s criteria [14, 15] and 13 (9 males) were prepubertal. All but 2 prepubertal patients (1 male) showed signs of virilization with abnormally advanced pubic hair growth or genital development (Table 1). Hypertension in the pediatric and adult subjects was defined as previously described [16, 17].

### Diagnosis of CD

Diagnosis of CD was made on the basis of loss of cortisol circadian rhythm, elevated sleeping midnight serum cortisol >50 nmol/l, detectable plasma ACTH, failure to suppress cortisol to <50 nmol/l during a low-dose dexamethasone suppression test (LDDST) and exaggerated increase of serum cortisol during a human-sequence corticotrophin-releasing hormone (1 μg/kg IV CRH) test [1]. Suppression of cortisol by >50 % during high-dose dexamethasone suppression test (HDDST), also consistent with CD, was a further feature [18].

### Pituitary imaging

Magnetic resonance imaging (MRI) was performed at 1.5T preoperatively in 20 patients as previously described [19]. One patient, investigated in 1986, had a pituitary CT scan [19].

### Bilateral simultaneous inferior petrosal sinus sampling for ACTH (BSIPSS)

BSIPSS was performed without general anesthetic or sedation as previously described [18, 20]. Central ACTH secretion was confirmed by an inferior petrosal sinus (central) to peripheral ACTH ratio ≥3.0 (IPS/P) after administration of 100 μg IV CRH. Lateralization of ACTH secretion was defined as an inter-petrosal sinus ACTH gradient (IPSG) of ≥1.4 [20].

### Hormone assays

Serum cortisol, T4 (free and total), TSH, PRL, LH, FSH, GH, ACTH and testosterone were determined as previously described [19].

### Auxology and puberty staging

Standardized anthropometric techniques were used to measure height, weight and height velocity [21]. Target height (TH; in cm) was calculated using the formula: mother’s height + father’s height + 13 divided by 2 for boys, mother’s height + father’s height –13 divided by 2 for girls, and was expressed as SDS [4]. Adult height (AH) was defined as height when measurements over 12 months differed by <1 cm. BMI and height were converted to standard deviation scores (SDS) as previously described [22]. Pubertal development was staged according to the criteria of Tanner and testicular volumes were measured using a Prader orchidometer [14, 15].

### Definitive therapy and remission of CD

Transsphenoidal pituitary exploration (TSS), aimed at selective microadenomectomy, was performed in all patients by two neurosurgeons, FA or IS. Biochemical remission after TSS was defined as serum cortisol <50 nmol/l on at least 3 mornings during the immediate post-operative period [1]. Patients in whom post-operative remission was not achieved (n = 5) had second-line therapy with external pituitary irradiation (RT) [18]. Biochemical remission following RT was defined as mean serum cortisol <150 nmol/l on a 5-point day-curve and midnight cortisol of <50 nmol/l [18]. The RT was delivered at a mean interval of 85.8 days (23–177) after unsuccessful TSS. Recovery of serum cortisol post-TSS or RT was defined as mean serum cortisol 150–300 nmol/l on a 5-point day curve [18]. Post-operatively, patients with cortisol <50  nmol/l were commenced on replacement hydrocortisone therapy. The HPA axis was reassessed at regular intervals for recovery as previously described [23].

### Recurrence of CD and Nelson syndrome

Recurrence of CD was defined as the presence of biochemical features of CD i.e. increased midnight sleeping cortisol, lack of suppression of cortisol during LDDST and increased 24 h urinary free cortisol values, which recurred after definitive therapy that resulted in clinical remission. Nelson syndrome was defined as enlarging pituitary corticotropinoma with elevated and rising ACTH levels following bilateral adrenalectomy [24].

### Assessment of GH secretion

GH secretion was assessed as follows: insulin tolerance test (ITT, 0.15 U/kg insulin IV), glucagon stimulation (15 mcg/kg IM) and arginine test (0.5 g/kg l-arginine monohydrochloride IV). GH deficiency (GHD) was defined as peak GH <7 μg/l during the stimulation test [4, 8]. GH secretion was first assessed during remission following definitive treatment of CD (short-term assessment) and again after completion of linear growth (long-term assessment). GHD in adolescents and adults was defined according to consensus publications [25, 26].

### Statistical analysis

Statistical analysis was performed using IBM SPSS Statistics 23. The paired-samples *T* Test was used to determine significant differences between height SDS, BMI SDS, target height SDS and height SDS at diagnosis and follow-up.

## Results

### Diagnostic investigations

Mean sleeping serum cortisol at midnight was 505.1 nmol/l (142–1082) and 09.00 h ACTH 42.4 ng/l (13–96, normal 10–50). All patients had a LDDST and 18/21 (86 %) showed inadequate cortisol suppression. HDDST was performed in 12 (57 %) patients and 9/12 (75 %) suppressed 09.00 h cortisol at 48 h to <50 nmol/l. The CRH test was performed in all patients and induced a mean cortisol increase of 90.5 %: in 19/21 (90 %), the cortisol rise during the CRH test was >20 %.

MRI or CT scans of the hypothalamo-pituitary region showed abnormalities consistent with a pituitary adenoma in 9/21 (43 %). Concordance between the site of the adenoma on imaging and at surgery was 3/21 (14.3 %). Nineteen (91 %) patients had BSIPSS, which showed lateralization of ACTH secretion in 14 (73.7 %; 7 right, 7 left): concordance between lateralization of the tumor on BSIPSS and during surgery was 14/19 (74 %) with 87 % (13/15) concordance in the remission patients vs 25 % (1/4) in the non-remission patients. The results of the biochemical and imaging investigations are shown in Tables 1 and 2.

**Table 2:** Details of pituitary imaging, BSIPSS, pituitary histology, definitive treatment and outcome

Pt no.	BSIPSS	MRI/CT	Adenoma position at TSS	Pituitary histology	09.00 h Cortisol nmol/L post-TSS (day)	Definitive treatment	Interval (yrs) to post-RT remission	Time of recovery of pituitary-adrenal axis (yr) post-TSS or RT	Time of recurrence (yrs) post- definitive treatment	Long-term outcome
1	ML	R	R	N	719 (7)	TSS^a^ + TSS + BA	–	–	No	Nelson’s syndrome (0.6 years post BA)
2	–	ML	N (biopsy)	N	269 (10)	TSS + RT	0.9	1.2	No	Remission
3	–	ML	R	Adenoma	630 (4)	TSS + RT	0.3	1.1	No	Remission
4	L	N	R	N	600 (5)	TSS + RT	0.1	0.4	Yes (7.6)	Abnormal cortisol circadian rhythm—no treatment
5	ML	L	L	N	612 (6)	TSS + RT	0.4	0.6	No	Remission
6	L	N	L	N	382 (8)	TSS + RT	2.2	–	No	BA for ACTH-independent cortisol secretion (3.9 years post-RT)—remission
7	L	ML/R	ML	N	43 (5)	TSS	–	–	Yes (2.0)	TSS—no remission, RT—remission (1.4 years post RT)
8	R	N	R	Adenoma	<50 (4)	TSS	–	No recovery	No	Remission
9	L	R	L	Adenoma	<50 (3)	TSS	–	12.0	No	Remission
10	R	N	R	Adenoma	<50 (6)	TSS	–	–	No	Remission
11	R	N	R	N	<50 (5)	TSS	–	Yes	No	Remission
12	L	R	L	N	<50 (5)	TSS^a^ + TSS	–	1.8	No	Remission
13	ML	L	ML/R	N	<50 (6)	TSS	–	0.7	No	Remission
14	R	N	R	Adenoma	<50 (2)	TSS	–	2.3	No	Remission
15	L	ML	ML	Adenoma	<50 (2)	TSS	–	–	Yes (6.0)	RT—no remission, metyrapone—no remission, pasireotide-remission (3.0 years post RT)
16	L^b^	N	L	Adenoma	36 (8)	TSS	–	1.7	No	Remission
17	R^b^	N	R	Adenoma	22 (5)	TSS	–	–	No	Remission
18	R	N	R	Adenoma	44 (5)	TSS	–	2.1	No	Remission
19	ML	N	ML	Adenoma	<50 (7)	TSS	–	4.0	No	Remission
20	R	N	R	Adenoma	20 (4)	TSS	–	1.45	No	Remission
21	ML	N	Diffuse adenoma	Adenoma	21 (5)	TSS	–	No recovery	No	Remission

*BSIPSS* bilateral simultaneous inferior petrosal sinus sampling, *IPS/P* interpetrosal sinus ACTH gradient (≥1.4 suggests lateralisation) (2), *L* left-sided lateralisation/adenoma position, *R* right-sided lateralisation/adenoma position (^b^performed under general anesthesia); *ML* no lateralisation/midline position, *N* normal, *TSS* transsphenoidal surgery (^a^performed at another centre), *RT* external beam pituitary radiotherapy, *BA* bilateral adrenalectomy

### Transsphenoidal surgery (TSS)

Transnasal endoscopic TSS (performed by surgeons IS, GA) was undertaken in 3 patients (Patients 17, 21 and the second procedure for Patient 14). A translabial transsphenoidal approach was used in the remaining surgical procedures (performed by surgeon FA). 14 patients (67 %) had biochemical remission after a single TSS procedure and one patient after two consecutive TSS procedures (Patient 12), the first being performed at another center. The overall rate of remission following TSS in our center was 71 %.

Five patients (Patients 2–6; 24 %) had elevated cortisol post-operatively with values ranging from 269 to 900 nmol/l (mean 478 nmol/l) indicating lack of remission. In all cases, remission was achieved following second-line RT, with a mean interval between RT and remission of 0.8 years (0.1–2.2). Patient 1 who had two consecutive unsuccessful TSS procedures underwent bilateral adrenalectomy (BA) because of severe, acute psychosis [27]. Details of the treatment modalities are shown in Table 2.

### Pituitary histology

In 20/21 (95 %) patients a pituitary microadenoma was identified at surgery, in all patients in remission after a single TSS procedure, and in 4/5 (80 %) in whom TSS was unsuccessful (Table 2). In 12/21 (57 %) patients, positive histology confirmed a corticotrope adenoma. Positive histology was present in 11/15 (73 %) patients with post-operative remission and 1/6 (17 %) patients without remission (Table 2).

### Recurrence rates and management

Clinical and biochemical recurrence of hypercortisolemia was seen in 2 patients (Patients 7 and 15) (10 %) and mild biochemical abnormalities, suggestive of subclinical recurrence, in Patient 4 (Table 2). The features in Patient 7 were weight gain and growth arrest, increased midnight cortisol and MRI evidence of a microadenoma, but normal cortisol suppression during LDDST 2.0 years after initial remission. A second TSS was unsuccessful, but biochemical remission was achieved 1.4 years after pituitary RT. Patient 15 relapsed 6 years after successful TSS, and subsequent RT failed to induce remission. The patient refused bilateral adrenalectomy and is being managed with pasireotide, which is controlling the hypercortisolemia. Patient 4 had consistently raised midnight cortisol 7.6 years after RT. There were no signs of hypercortisolemia and LDDST showed adequate cortisol suppression. This patient is on no treatment with no progression for 10 years.

### Other complications of definitive treatment

Patient 6 developed autonomous (ACTH-independent) adrenal cortisol secretion following TSS + RT (3.9 years post-RT) and was cured following bilateral adrenalectomy. At 1.5 years post-adrenalectomy, he had normal ACTH secretion and no evidence of a pituitary adenoma on pituitary MRI. Patient 1 had elevated and increasing ACTH (333 ng/L, normal 10–50) associated with MRI enlargement of the pituitary adenoma (3–7 mm diameter) and underwent pituitary radiotherapy 0.6 years following bilateral adrenalectomy. The ACTH increased to 1253 ng/L approximately 0.5 years after completion of RT. At latest assessment, 2.5 years post-RT, the adenoma measures 8.2 mm and ACTH is 373 ng/L.

### Anterior and posterior pituitary function

#### GH secretion

Short-term GH assessment of was performed in 19 patients at a mean interval of 0.5 years (0.1–1.4) after the last definitive treatment for CD, TSS (n = 15 patients) and RT (n = 4 patients) (mean peak GH 6.4 μg/l, range < 0.5–18.3). GHD was present in 14/19 (81 %) (mean peak GH 3.1 μg/l, range < 0.5–6.9), 79 % after TSS and 21.5 % after RT (Table 3). GH therapy was administered in 14 patients (77.8 % of all tested), 11 (73 %) after TSS and 3 (75 %) after RT, and continued in 12 until adult or near-adult height.

**Table 3:** Short- and long-term assessment of growth hormone secretion and growth hormone treatment

		Short -term assessment						Long-term assessment				
Pt no.	Definitive treatment	Time interval (yr)	GH test	GH peak (μg/l)	GHD	GH treatment	GH treatment (yr)	Other CD treat-ment	GH test	GH peak (μg/l)	Time interval (yr)	GHD
1	2 TSS + BA	–	–	–	–	No	–	RT	–	–	–	–
2	TSS + RT	0.7	Glucagon	6.9	Yes	Yes	8.0 (AH)	–	ITT	5.7	9.6	No
3	TSS + RT	0.7	Glucagon	6.4	Yes	Yes	3.5 (AH)	–	ITT	11.4	8.6	No
4	TSS + RT	1.4	ITT	16.5	No	Yes	1.8 (AH)	–	ITT	19.4	7.6	No
5	TSS + RT	0.3	ITT	<1.0	Yes	Yes	7.1 (AH)	–	–	–	–	–
6	TSS + RT	–	–	–	–	No	–	BA	–	–	–	–
7	TSS	0.2	ITT	12	No	Yes (+Letrozole)	1.5 (AH)	TSS + RT	–	–	–	–
8	TSS	0.7	Arginine	1.1	Yes	Yes	9.9 (LA)	–	ITT	0.9	10.0	Yes
9	TSS	0.7	ITT	18.3	No	No	–	–	–	–	–	–
10	TSS	–	ITT	3.8	Yes	Yes	–	–	ITT	10.1	17.0	No
11	TSS	0.2	ITT	4.0	Yes	Yes	7.7 (AH)	–	ITT	8.2	8.1	No
12	2 TSS	0.2	ITT	<0.5	Yes	Yes	10.7 (AH)	–	ITT	0.4	7.5	Yes
13	TSS	0.1	ITT	5.5	Yes	No	–	–	–	–	–	–
14	TSS	0.1	ITT	3.5	Yes	Yes	12.5 (AH/LA)^a^	–	–	–	–	–
15	TSS	–	–	–	Yes	Yes	–	RT	–	–	–	–
16	TSS	0.3	Glucagon	16.1	No	Yes	3.4 (LA)	–	Glucagon	0.4	3.8	Yes
17	TSS	0.8	Glucagon	9.5	No	No	–	–	–	–	–	–
18	TSS	0.5	ITT	2.6	Yes	Yes	1.6 (AH)	–	–	–	–	–
19	TSS	0.8	ITT	1.6	Yes	Yes (+GnRHa)	3.4	–	ITT	0.9	4.6	Yes
20	TSS	0.5	Glucagon	5.3	Yes	No	–	–	–	–	–	–
21	TSS	0.9	ITT	0.6	Yes	Yes	4.3 (LA)	–	–	–	–	–

*TSS* transsphenoidal surgery, 2 *TSS* two surgical procedures undertaken (see Table 2), *RT* external beam pituitary radiotherapy, *BA* bilateral adrenalectomy, *Time interval* time interval between the last definitive treatment inducing remission and GH testing, *ITT* insulin tolerance test, *Time interval* the interval between definitive treatment and GH testing, *GnRHa* gonadotrophin releasing hormone analogue, *AH* adult height, *LA* latest assessment

^a^GH therapy ongoing until peak bone mass achieved

Long-term GH assessment was performed in 9 patients at a mean interval of 8.5 years (3.8–17.0) after the last definitive treatment, TSS (n = 6 patients) and RT (n = 3 patients) (mean peak GH 6.4 μg/l, range 0.4–19.4). Long-term GHD (mean peak GH 0.7 μg/l, range 0.4–0.9) was present in 4/9 investigated (44 % of all tested): 67 % after TSS and 33 % after RT (Table 3). GH therapy was continued in patient 14 who had GHD post-TSS and has completed his linear growth. This patient has not been retested for GHD but presented with vertebral fractures and GH therapy is on-going until he achieves peak bone mass.

#### Gonadotropin secretion

At latest assessment, 14 patients (8 males; 66.7 %) had completed and 7 patients (5 males; 33.3 %) were undergoing pubertal development. Nine patients (7 males; 42.9 %) had delayed onset or slow progression of puberty. Six required hormonal replacement (Table 4). Five males received testosterone or FSH + HCG therapy (Patients 5, 8, 12, 14, 21). Four (19 %) (Patients 1, 14, 15, 21) had evidence of long-term hypogonadotropic hypogonadism requiring replacement therapy as adults. Three female patients have had children (Table 4).

**Table 4:** Anterior and posterior pituitary hormone deficiencies following treatment for paediatric CD

Pt no.	CD Therapies	Puberty features	Pubertal induction	Additional long-term pituitary deficiencies					
				Gonadotrophin	AVP	TSH	ACTH	PRL	Other features
1	2 TSS + BA, RT	Slow progression, late menarche at 16.0 years and poor breast development.	+ (Oral oestrogen for 0.6 years)	+ (HRT since aged 20 years)	–	–	–	–	Breast Augmentation aged 17 years
2	TSS + RT	no	–	–	–	–	–		
3	TSS + RT	no	–	–	–	–	–	–	
4	TSS + RT	–	–	–	–	–	–	–	
5	TSS + RT	Slow progression	+ (Testosterone for 2.3 years)	–	–	–	–	–	
6	TSS + RT, BA	Slow progression	–	–	–	–	–		
7	2TSS + RT	Slow progression	–	–	–	–	–	–	Poor semen quality aged 17 years
8	TSS	Delayed and slow progression	+ (Testosterone for 1.2 years)	–	–	+	+	+	
9	TSS	–	–	–	–	–	–		PCOS 3 children
10	TSS	–	–	–	–	–	–		HRT aged 37 years 3 children
11	TSS	Late menarche (17.6 years)	–	–	–	–	–	–	Low libido 1 child
12	2TSS	Delayed onset	+ (Testosterone for 0.3 years)	–	–	–	–	–	
13	TSS	–	–	–	–	–	–	–	
14	TSS	Pubertal arrest	+ Testosterone since aged 16years	+	–	–	–		
15	TSS + RT	–	–	+	+	–	–		
16	TSS	–	–	–	–	–	–		
17	TSS	–	–	–	–	–	–		
18	TSS	–	–	–	–	–	–	High (737, rr 0–496)	
19	TSS	–	–	–	–	–	–		
20	TSS	–	–	–	–	–	–	–	
21	TSS (diffuse tumour)	HH	+ (HCG, FSH)	+ (Testogel)	+	+	+	+	Normal semen analysis

*HH* hypogonadotropic hypogonadism, + deficiency present, - deficiency absent, *AVP* vasopressin, *TSH* thyroid stimulating hormone deficiency, *ACTH* adrenocorticotropin deficiency, *PRL* prolactin deficiency

#### TSH secretion

TSH deficiency was seen in 2 patients (9.5 %). Both had a single TSS procedure (Patients 8, 21) and had associated pituitary hormone deficiencies (Table 4).

#### ACTH secretion

The mean time interval between recovery of the pituitary-adrenal axis following TSS (8 patients) was 3.2 years (0.7–12.0 years), and following RT (4 patients) 0.8 years (0.4–1.2 years). At latest assessment, 2 patients (9.5 %) had persistent ACTH deficiency (Patients 8, 21) requiring daily hydrocortisone replacement therapy (Table 4): both had one TSS procedure and had additional anterior pituitary defects.

#### AVP secretion

Seven patients (33.3 %) developed central diabetes insipidus (DI) post-TSS. AVP secretion recovered in 5/7 (71.4 %) patients, 4 in the immediate postoperative period. One patient required desmopressin therapy for 6 years. Two patients (10 %) had permanent DI associated with additional anterior pituitary defects.

#### Long-term multiple versus single pituitary hormone deficiencies

At latest assessment, 5 patients had a single pituitary hormone deficiency. GHD was the most frequent isolated long-term deficiency, occurring in 4 (19.0 %) and gonadotropin deficiency in 1 (5 %) **(**Table 3). Four patients had more than one anterior pituitary hormone deficiency (19 %) 3 after TSS (Patients 8, 14 and 21) and 1 following RT (Patient 15) (Table 4). Patient 8 had GH, gonadotropin, TSH,ACTH and prolactin deficiencies. Patient number 21 had GH, TSH, gonadotropin, ACTH, prolactin and AVP deficiencies. Patient number 14 had gonadotropin and GH deficiencies and patient 15 had gonadotropin and AVP deficiencies. Therefore, in total, 43 % patients had long-term pituitary deficiencies but only 5 of these (24 % of the whole cohort) receive on-going replacement therapies.

### Linear growth

**Fig. 1:**
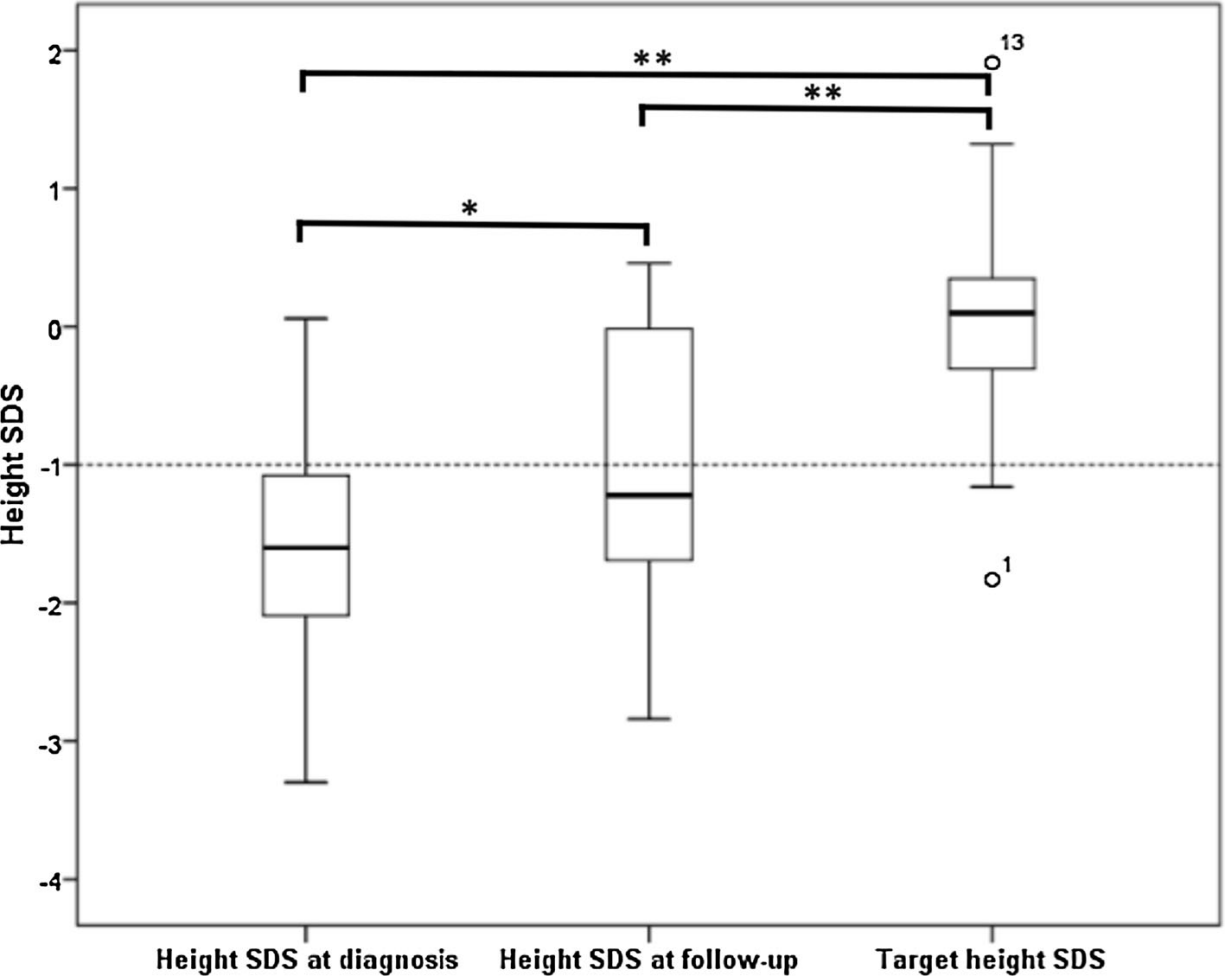
Height SDS at diagnosis, latest assessment and target height SDS (n = 15). Boxplot graph of the 15 patients with follow-up and target height growth data. Box, interquartile range (*lower quartile*, *median* and *upper quartile*); whiskers, maximum and minimum values; outliers represented with *circle* and patient number. *p = 0.033, **p = 0.000

At diagnosis, mean height SDS for the whole cohort was −1.5 (0.1 to −3.3). Mean height SDS at diagnosis for the 15 patients with FU height data was −1.6 (0.1 to −3.3) and the height SDS at adult height (AH) (n = 8) or latest assessment (LA) (n = 7) was mean −0.99 (0.46 to −2.84) at a mean 6.7 years (2.6–16.6) post-definitive therapy and 7.1 years (2.7–17.3) post-diagnosis. Therefore, height SDS at follow-up (AH or LA) had increased compared to diagnosis (mean change +0.60; range −0.79–2.90 SDS) (p = 0.033) (Fig. 1).

Mean target height (TH) SDS for the 15 patients with follow-up data was +0.03 (−1.83–1.91) and differed from the height SDS at diagnosis and AH or LA (p = 0.000) (Fig. 1). The difference between TH and AH/LA SDS (mean 1.01; range −0.17–2.44) was less than the difference between TH SDS and height SDS at diagnosis (1.61; range 0.22–3.4) (p = 0.033).

### BMI

**Fig. 2:**
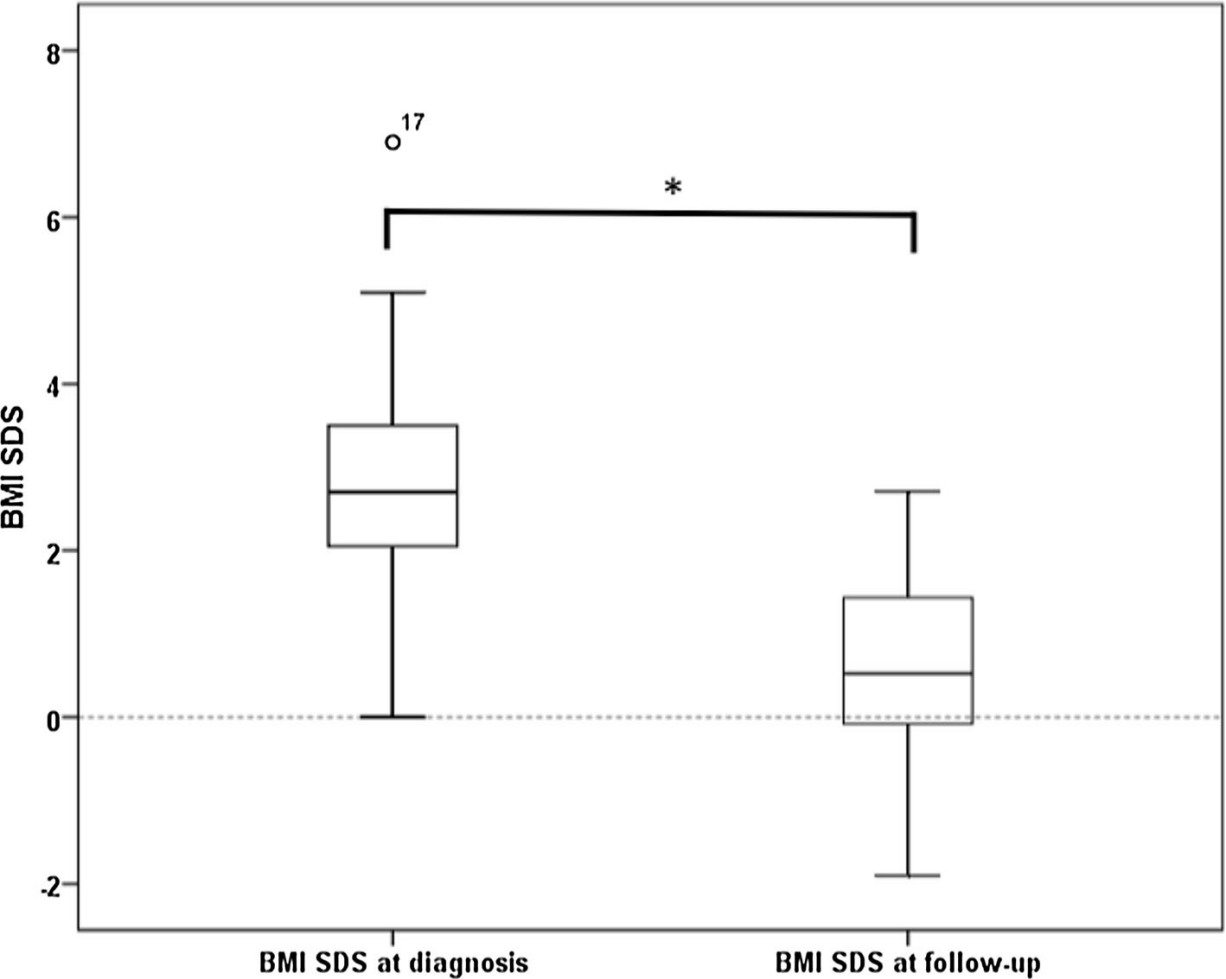
BMI SDS at diagnosis and follow-up (n = 15) Boxplot graph of the 15 patients with follow-up BMI data: box, interquartile range (*lower quartile*, *median* and *upper quartile*); whiskers, maximum and minimum values; outliers represented with *circle* and patient number. *p = 0.000

BMI SDS (n = 15) was lower at LA (mean 0.58; −1.9–2.71), compared to diagnosis mean BMI SDS 2.9 (0.1–6.9) (p = 0.000) (Fig. 2). The interval between definitive therapy and BMI LA was 6.7 years (range 2.6–16.6).

### Bone Mineral Density (BMD)

At diagnosis, 10 patients had BMD assessed by DEXA scan. L1-L4 areal BMD Z scores in 5 patients were mean −1.3 (−0.3 to −2.6) and mean L2-L4 areal BMD Z scores in 5 patients were −2.0 (−0.6 to −3.3). In 3/10 patients, femoral neck BMD was mean −1.7 (−1.0 to −2.1). The BMD Z-score at diagnosis was <−2 in 30 % of patients, −2 to <−1 in 50 % and −1 to <0 in 20 % patients. No patient had a BMD Z-score above 0.

Nine patients had follow-up (mean 5.2 years; 1.4–10.5) L2-L4 areal BMD mean Z score −0.5 (1.1 to −2.0). Mean areal femoral neck BMD Z score (5 patients) was 0.22 (2.46 to−0.9) after a mean follow-up interval of 5.83 years (1.4–10.5). The BMD Z-score at follow-up was < −2 in 20 % of patients, −2 to < −1 in 20 %, −1 to < 0 in 20 % and 0–2 in 40 % patients. Patients with lowest Z-score values at diagnosis were Patients 7 (L1-L4 BMD Z-score −2.6) and 14 who presented with vertebral fractures (L2-L4 aBMD Z-score −3.3) and Patient 18 (L2-L4 aBMD Z-score −3.0). These improved to −1.8, −2.0 and −1.0 at 1.8, 2.0 and 10 years, respectively.

### Blood pressure

At diagnosis, 9 (43 %) patients had hypertension. Blood pressure data were available in 9 patients at latest assessment. Three (33.3 %), had hypertension at a mean age 32.4 years (23.7–44.0), but were not prescribed antihypertensive medications.

### Psychiatric and cognitive problems

In total, 5 patients (24 %) had long-term psychiatric co-morbidities. Patient 17 had anxiety with challenging behavior. Patient 1 developed acute psychosis with self-injuries [27]. After resolution of the hypercortisolemia following bilateral adrenalectomy, the psychotic episode (and the associated cerebral volume loss on radiological imaging) resolved. She had prolonged hypercortisolemia before remission with social problems and low self-esteem. Following remission, she developed clinical depression treated with fluoxetine. Her symptoms improved after breast augmentation surgery and she is currently off anti-depressant therapy.

Three patients (14 %) reported problems with memory. Patient 4 had TSS + RT and reported transient problems with concentration following RT, but subsequently made good academic progress. Patient 5 (after RT) and Patient 21 (after TSS) had decreased short-term memory and concentration, causing impaired school performance. Patient 2 had occasional post-treatment mood swings.

## Discussion

CD is rare in childhood and a limited number of centers have the capacity to manage this condition comprehensively. Close collaboration with adult colleagues with experience of CD is beneficial for pediatric management [1]. The presenting features may differ in children compared to adults, with growth arrest and rapid weight gain being the main presenting features [1]. The signs and symptoms at diagnosis in our cohort of patients are consistent with reports from other centers [10, 13].

Transsphenoidal surgery, performed by pituitary surgeons with experience in children, is effective and safe first-line treatment [1, 10]. Reported rates of remission after TSS vary from 60 to 98 % in different studies [1], due largely to the lack of agreement on definition of post-operative remission. In all reports of pituitary surgery, a proportion of patients do not achieve biochemical remission and this was also the case in our series.

Identification of the corticotrope adenoma during surgery and its histological confirmation are positive predictors of remission [10, 13, 23, 28]. Our series confirmed this observation, with an adenoma identified during surgery in all patients in remission after TSS. One factor reported to be associated with a higher prevalence of recurrence of CD is a younger age (<20 years) at diagnosis [10, 12]. We found no association between recurrence and age. Patients in whom CD recurred had a shorter mean duration of symptoms, i.e. 0.9 years, compared with 1.9 years in subjects remaining in remission. There was no relationship between severity of clinical symptoms or biochemical abnormalities and recurrence.

Recurrence rates after remission of pediatric CD vary considerably from 8 % to > 40 % [10–12]. Even though relapse is usually expected during the first 5 years following definitive treatment, it may occur later [29] and the percentage of relapsed cases increases with length of observation [23]. In our series, mean follow-up was more than 10 years. We report a small but important prevalence of recurrence, demonstrating that life-long follow-up is required, and this is consistent with data from long-term follow-up in adult patients with CD (24).

Following successful TSS, most patients showed recovery of the pituitary-adrenal axis. Similar results were reported by Devoe et al. [8]. Time to recovery of the axis has been considered to be a predictive factor for CD recurrence in adults and in children [10, 13, 23]. Unfortunately, in our series data for recovery of the axis were not complete in the patients with recurrence; however, no patient in remission after TSS had recovery of the axis at less than 0.65 years after pituitary surgery.

At final evaluation, single pituitary hormone deficiencies were seen in 5 patients (24 %). Most common was GH deficiency (GHD), which is well-recognized following TSS [30] and pituitary irradiation [9, 18]. Additional pituitary deficiencies were rare. When these occurred they were associated with other anterior and posterior pituitary defects. The percentage of endocrine deficits in our series is higher than reported in other pediatric CD studies [8, 31]. The reason for this is unclear, but may be due to the fact that these patients have more active on-going surveillance.

Growth is frequently compromised in pediatric CD [1, 4, 32]. Even after successful treatment, children often did not attain the required catch-up growth to reach their target height and adult height was subnormal [32]. Following TSS and radiotherapy, GHD is frequently present [8, 32]. In our series, short-term GHD was seen in the majority of tested subjects, but following long-term retesting, GH secretion had recovered in a significant proportion of patients (71 %). Experience in our center has shown that hGH therapy can benefit children with suboptimal post-remission catch-up growth. Our advice, for early GH testing and initiation of hGH therapy when indicated, has recently been endorsed [5]. Vertebral bone mineral density (BMD) is more severely affected than femoral BMD in children with CD and is independent of the degree or duration of hypercortisolism [33]. In our series, mean lumbar spine BMD was reduced at diagnosis and improved at follow-up. Complete reversal to normal bone mass was not seen in all patients, suggesting some individuals may be at future risk of osteopenia.

Pubertal development is disturbed in many patients presenting with CD [1, 6]. In our study many subjects had disturbed timing or progression through puberty requiring sex steroid replacement due to the suppressive effect of hypercortisolemia on gonadotropins [6]. Close follow-up of pubertal development and early identification of pituitary–gonadal axis deficiency following remission of pediatric CD is important.

BMI is expected to decrease in patients with biochemical remission after definitive therapy, but may not decrease to normal values [4, 34] with persistence of central obesity [35]. In our series, mean BMI SDS decreased significantly during remission towards normalization in longer observation periods.

Psychological problems have been recognized with long-standing hypercortisolemia in the pediatric age range [36]. Many of our patients had emotional lability and fatigue at diagnosis, and two had more severe problems. In adults following treatment for CD, it is expected that psychiatric symptoms would resolve, although some disturbances might persist after cure [37]. The more severe psychiatric and behavioral symptoms at diagnosis improved significantly after cure. Hypercortisolemia may induce atrophic changes in the CNS, with volume loss in the temporal lobe, amygdala and the hippocampus [38, 39]. These effects might contribute to the behavioral and cognitive changes seen in adult and pediatric CS patients [39]. One small pediatric study reports declining cognitive function 1 year after cure of CS even after regaining lost brain volume [36]. In our series, 3 patients reported cognitive problems after treatment for CD, mostly with short-term memory.

In experienced hands, the prognosis for pediatric CD is good with various therapeutic options available, ranging from transsphenoidal microadenomectomy to bilateral adrenalectomy. As the majority of corticotrope adenomas in children and adolescents are microadenomas, optimal surgical care consists of tumor resection with maintenance of pituitary tissue. Our current long-term experience indicates that the diagnostic assessment and management of children with CD is broadly similar to that seen in adult patients, but growth is an important additional parameter, which must be assessed and treated appropriately. GHD can be managed with hGH replacement. There are long-term improvements in growth, weight, bone density and mental state, although in many cases they may not return entirely to normal. Recurrence of hypercortisolemia following successful post-TSS or post-RT-induced remission is unusual, but does occur as is also seen in adults. In our series, no key factors predictive of recurrence were identified. There are clear indications that life-long endocrine surveillance of pediatric patients, in whom remission has been induced by pituitary surgery or radiotherapy, remains mandatory.
